# Glycerol or crude glycerol as substrates make *Pseudomonas aeruginosa* achieve anaerobic production of rhamnolipids

**DOI:** 10.1186/s12934-021-01676-2

**Published:** 2021-09-23

**Authors:** Feng Zhao, Yuting Wu, Qingzhi Wang, Mengyao Zheng, Qingfeng Cui

**Affiliations:** 1 School of Life Sciences, Qufu Normal University, Qufu, 273165 Shandong China; 2 Research Institute of Petroleum Exploration and Development (Langfang), Langfang, 065007 Hebei China

**Keywords:** Rhamnolipids, *Pseudomonas aeruginosa*, Anaerobic production, Glycerol, Strain specificity

## Abstract

**Background:**

The anaerobic production of rhamnolipids is significant in research and application, such as foamless fermentation and *in situ* production of rhamnolipids in the anoxic environments. Although a few studies reported that some rare *Pseudomonas aeruginosa* strains can produce rhamnolipids anaerobically, the decisive factors for anaerobic production of rhamnolipids were unknown.

**Results:**

Two possible hypotheses on the decisive factors for anaerobic production of rhamnolipids by *P. aeruginosa* were proposed, the strains specificity of rare *P. aeruginosa* (hypothesis 1) and the effect of specific substrates (hypothesis 2). This study assessed the anaerobic growth and rhamnolipids synthesis of three *P. aeruginosa* strains using different substrates. *P. aeruginosa* strains anaerobically grew well using all the tested substrates, but glycerol was the only carbon source that supported anaerobic production of rhamnolipids. Other carbon sources with different concentrations still failed for anaerobic production of rhamnolipids by *P. aeruginosa*. Nitrate was the excellent nitrogen source for anaerobic production of rhamnolipids. FTIR spectra analysis confirmed the anaerobically produced rhamnolipids by *P. aeruginosa* using glycerol. The anaerobically produced rhamnolipids decreased air-water surface tension to below 29.0 mN/m and emulsified crude oil with EI_24_ above 65%. Crude glycerol and 1, 2-propylene glycol also supported the anaerobic production of rhamnolipids by all *P. aeruginosa* strains. Prospects and bottlenecks to anaerobic production of rhamnolipids were also discussed.

**Conclusions:**

Glycerol substrate was the decisive factor for anaerobic production of rhamnolipids by *P. aeruginosa*. Strain specificity resulted in the different anaerobic yield of rhamnolipids. Crude glycerol was one low cost substrate for anaerobic biosynthesis of rhamnolipids by *P. aeruginosa*. Results help advance the research on anaerobic production of rhamnolipids, deepen the biosynthesis theory of rhamnolipids and optimize the anaerobic production of rhamnolipids.

## Background

Rhamnolipids is the most extensively studied biosurfactants nowadays [1–3]. Due to its relatively high yield and good activity, rhamnolipids has great application potential in enhanced oil recovery, bioremediation, agriculture, food, cosmetic, and other fields [3–6]. The anaerobic production of rhamnolipids is significant in research and application, such as in situ production of rhamnolipids in the anoxic environments and foamless fermentation.

Oil reservoirs, deep soil and sediments are anoxic environments. The anaerobic production of rhamnolipids can achieve in situ production of rhamnolipids in such environments [7–9]. Rhamnolipids is a good oil displacement agent for enhanced oil recovery due to its excellent emulsifying activity to crude oil and surface activity to reducing interfacial tension of water/oil/rock [10]. In situ production of rhamnolipids in oil reservoirs is more cost-effective and easy to operate for enhanced oil recovery [11–13]. Producing rhamnolipids anaerobically by microorganisms can achieve in situ production of rhamnolipids in anoxic oil reservoirs.

The foam problem in aerobic fermentation has been perplexing the rhamnolipids production [14]. During the aerobic production of rhamnolipids, foam is minute bubbles mainly formed by rhamnolipids liquid membrane filling air. Adding defoamer can just solve the foam problem at some extent [14, 15]. Moreover, rhamnolipids fermentation without aeration can avoid the foam formation and develop foamless fermentation. Anaerobic production of rhamnolipids can achieve the foamless fermentation of rhamnolipids [7].

Rhamnolipids-producing bacteria mainly including *Burkholderia* sp. and *Pseudomonas* sp. [1, 3]. At present, *Pseudomonas aeruginosa* is considered to be the most productive rhamnolipids producer [1, 3], which makes *P. aeruginosa* become the research focus. *P. aeruginosa* is one of facultative anaerobic bacteria [7, 16, 17]. Although *P. aeruginosa* can grow at both aerobic and anaerobic conditions, studies on rhamnolipids production by *P. aeruginosa* were focused on the aerobic conditions [7]. Only a few studies reported that some rare *P. aeruginosa* strains can produce rhamnolipids anaerobically, such as *P. aeruginosa* ATCC10145, *Pseudomonas* sp ANBIOSURF-1, *P. aeruginosa* SG [8, 9, 13, 18–21].

Can other *P. aeruginosa* strains produce rhamnolipids anaerobically? The decisive factors for anaerobic production of rhamnolipids were still unknown. Revealing the decisive factors are helpful to advance the research on anaerobic production of rhamnolipids.

In this study, two possible hypotheses on the decisive factors for anaerobic production of rhamnolipids were proposed, the strains specificity of *P. aeruginosa* (hypothesis 1) and the effect of specific substrates (hypothesis 2). Using diverse substrates, anaerobic growth and rhamnolipids production of different *P. aeruginosa* strains were investigated. Three *P. aeruginosa* strains isolated from different sources were used. Diverse carbon sources and nitrogen sources were tested for anaerobic production of rhamnolipids. Anaerobically produced rhamnolipids was confirmed by Fourier transform infrared (FTIR) spectra analysis. The physicochemical properties of rhamnolipids products were also evaluated. Prospects and bottlenecks of anaerobic production of rhamnolipids were also discussed. Results would deepen the biosynthesis theory of rhamnolipids and guide the optimization process for anaerobic production of rhamnolipids.

## Materials and methods

### Strains and culture conditions

**Fig. 1:**
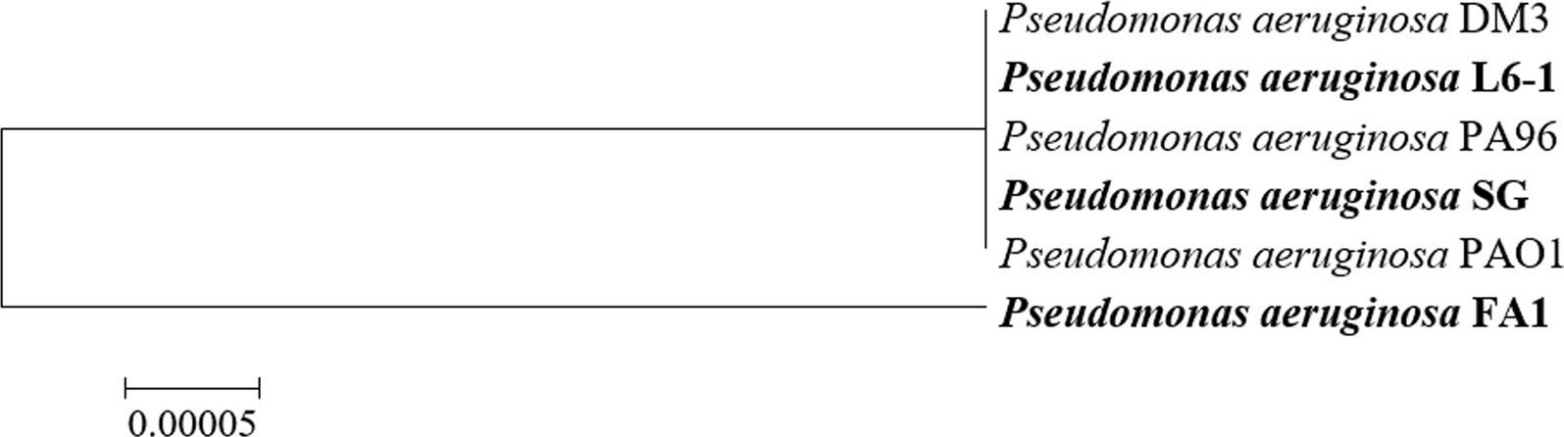
Neighbour-joining phylogenetic tree of three *P. aeruginosa* strains constructed by software Mega 5.0 based on a neighbour-joining analysis of 1000 resampled datasets. Bar, 0.00005 nucleotide substitutions per site

In this study, three *P. aeruginosa* strains (SG, L6-1 and FA1) isolated from different sources were investigated for rhamnolipids production. Strain *P. aeruginosa* SG and L6-1 were respectively isolated from production fluid of different oil reservoirs in Xinjiang Oilfield, China [8]. Strains *P. aeruginosa* FA1 were isolated from agricultural soil in Shandong province, China. The phylogenetic relationship of the three *P. aeruginosa* strains was shown in Fig. 1. *P. aeruginosa* SG was used as the positive control for anaerobic production of rhamnolipids using glycerol [8]. LB medium was used to prepare the seed culture at 35 °C and 180 rpm. For anaerobic cultivation, *P. aeruginosa* strains were cultured in 100 ml serum bottles containing 80 ml anaerobic medium. The inoculum amount was 3%. Briefly, the anaerobic medium was prepared as follows [8]. The medium was boiled for 15 min, and then 99.99% purity of N_2_ gas was injected into the boiling medium for 5 min to drive out oxygen. Then medium was sub-packaged in serum bottles when it was hot. Press the rubber plug and press the aluminum cap under N_2_ gas protection. After sterilization and cooling to 30 °C, deoxidizer Na_2_S·9H_2_O was added into the medium to a final concentration of 0.02% (w/v) [20]. The anaerobic fermentation experiments were performed at 35 °C and 50 rpm for 10 days. Three parallel experiments were set for each bacterial strain. The non-inoculated medium was used as the negative control.

### Analytical methods

Anaerobic culture samples were taken from serum bottles using sterile syringes. Bacterial biomass was represented using OD_600_ values of the anaerobic culture. Culture samples were centrifuged at 10,000*g* and 10 ℃ for 10 min. The surface tension of supernatant was measured by surface tensiometer (BZY-1, Shanghai Hengping Instrument and Meter Factory, Shanghai, China). The diameter of oil spreading circle formed by culture supernatant was determined as previous reported [22]. The used crude oil (7-Middle area, Xinjiang oilfield, China) has the density of 0.823 g/ml and the viscosity of 8.6 mPa·s at 30 ℃. Emulsification index (EI_24_) was measured to evaluate the emulsifying activity of anaerobically produced rhamnolipids [20]. EI_24_ values were calculated as the height of the emulsified layer (mm) divided by the total height of the liquid column (mm) and multiplied by 100.

### *P. aeruginosa* strains fed with different carbon sources

In this study, the commonly used carbon sources for aerobic production of rhamnolipids were investigated for anaerobic production of rhamnolipids. The selected carbon sources were glucose, glycerol, palmitic acid and soybean oil. The concentrations of carbon sources in the medium were all 40 g/l. The medium except carbon source contained 4 g/l of NaNO_3_, 4 g/l of K_2_HPO_4_·3H_2_O, 3 g/l of KH_2_PO_4_, 0.5 g/l of MgSO_4_·7H_2_O, 0.5 g/l of KCl, 0.5 g/l of NaCl, and 0.2 g/l of CaCl_2_·2H_2_O. After anaerobic cultivation, the OD_600_, surface tension and oil spreading activity were analyzed. Anaerobic production of rhamnolipids was defined as reducing the surface tension of anaerobic culture to lower than 35 mN/m and forming oil spreading circles with diameters greater than 15 mm.

### *P. aeruginosa* strains cultured using carbon sources with different concentrations

For the tested carbon sources f, using a single concentration maybe not persuasive. Glucose, glycerol, palmitic acid and soybean oil with concentrations of 15 g/l and 60 g/l were also studied for anaerobic production of rhamnolipids by three *P. aeruginosa* strains. The medium except carbon source contained 4 g/l of NaNO_3_, 4 g/l of K_2_HPO_4_·3H_2_O, 3 g/l of KH_2_PO_4_, 0.5 g/l of MgSO_4_·7H_2_O, 0.5 g/l of KCl, 0.5 g/l of NaCl, and 0.2 g/l of CaCl_2_·2H_2_O. After anaerobic cultivation, the surface tension and oil spreading activity of culture samples were analyzed.

### *P. aeruginosa* strains fed with different nitrogen sources

The common nitrogen nutrients, NH_4_Cl, NaNO_3_, yeast extract and peptone, were tested for the anaerobic production of rhamnolipids by *P. aeruginosa* strains. Strains *P. aeruginosa* SG was used as the positive control. The concentrations of nitrogen sources in the medium were all 4 g/l. The complex nitrogen sources of NaNO_3_ and yeast extract (3:7, 5:5, 7:3) were also tested for anaerobic production of rhamnolipids by *P. aeruginosa* strains. The amount of complex nitrogen sources was also 4 g/l. The medium except nitrogen source contained 40 g/l of glycerol, 4 g/l of K_2_HPO_4_·3H_2_O, 3 g/l of KH_2_PO_4_, 0.5 g/l of MgSO_4_·7H_2_O, 0.5 g/l of KCl, 0.5 g/l of NaCl, and 0.2 g/l of CaCl_2_·2H_2_O. The OD_600_ values, surface tension and oil spreading activity of culture samples were analyzed after anaerobic cultivation.

### Rhamnolipids extraction from the anaerobic culture

The anaerobically produced rhamnolipids were extracted as previous references described [9, 23]. Briefly, the cell free anaerobic culture broth was heated at 80 °C for 30 min to denature the extracellular proteins. Samples were centrifuged at 10,000*g* for 10 min. Using 6 mol/l of HCl-water solution, the pH value of samples was adjusted to 2.0. The organic solvent, chloroform and methanol (v/v, 2:1), was used for rhamnolipids extraction from aqueous phase. The rhamnolipids products were recovered from the organic phase using a rotary evaporator (50 rpm, 45 °C).

### FTIR spectra analysis

Anaerobically produced rhamnolipids was confirmed by FTIR spectra analysis. Anaerobically produced rhamnolipids from *P. aeruginosa* SG was used as the positive control. Briefly, 10 mg of rhamnolipids extract and 90 mg of KBr was mixed to make the translucent pellet with pressure of 25 Mpa for 25 s. A NICOLET 380 FTIR spectrometer was used to record the FTIR spectra of the translucent pellet with the wave number from 400 cm^− 1^ to 4000 cm^− 1^ [9, 20].

### Feasibility of glycerol intermediates and crude glycerol for anaerobic production of rhamnolipids

The possible glycerol intermediates, hydroxyacetone, 1, 2-propylene glycol and 1, 3-propylene glycol, were evaluated as carbon sources for anaerobic production of rhamnolipids by three *P. aeruginosa* strains. The concentration of glycerol intermediates was 40 g/l. The medium except carbon source contained 4 g/l of NaNO_3_, 4 g/l of K_2_HPO_4_·3H_2_O, 3 g/l of KH_2_PO_4_, 0.5 g/l of MgSO_4_·7H_2_O, 0.5 g/l of KCl, 0.5 g/l of NaCl, 0.2 g/l of and CaCl_2_·2H_2_O. Crude glycerol was also attempted for anaerobic production of rhamnolipids by *P. aeruginosa* strains. The tested crude glycerol containing 95% of glycerol. The concentration of crude glycerol in the medium was 40 g/l. After anaerobic cultivation, the OD_600_, surface tension and oil spreading activity of culture samples were analyzed.

## Results and discussion

### Anaerobic production of rhamnolipids using different carbon sources

**Fig. 2:**
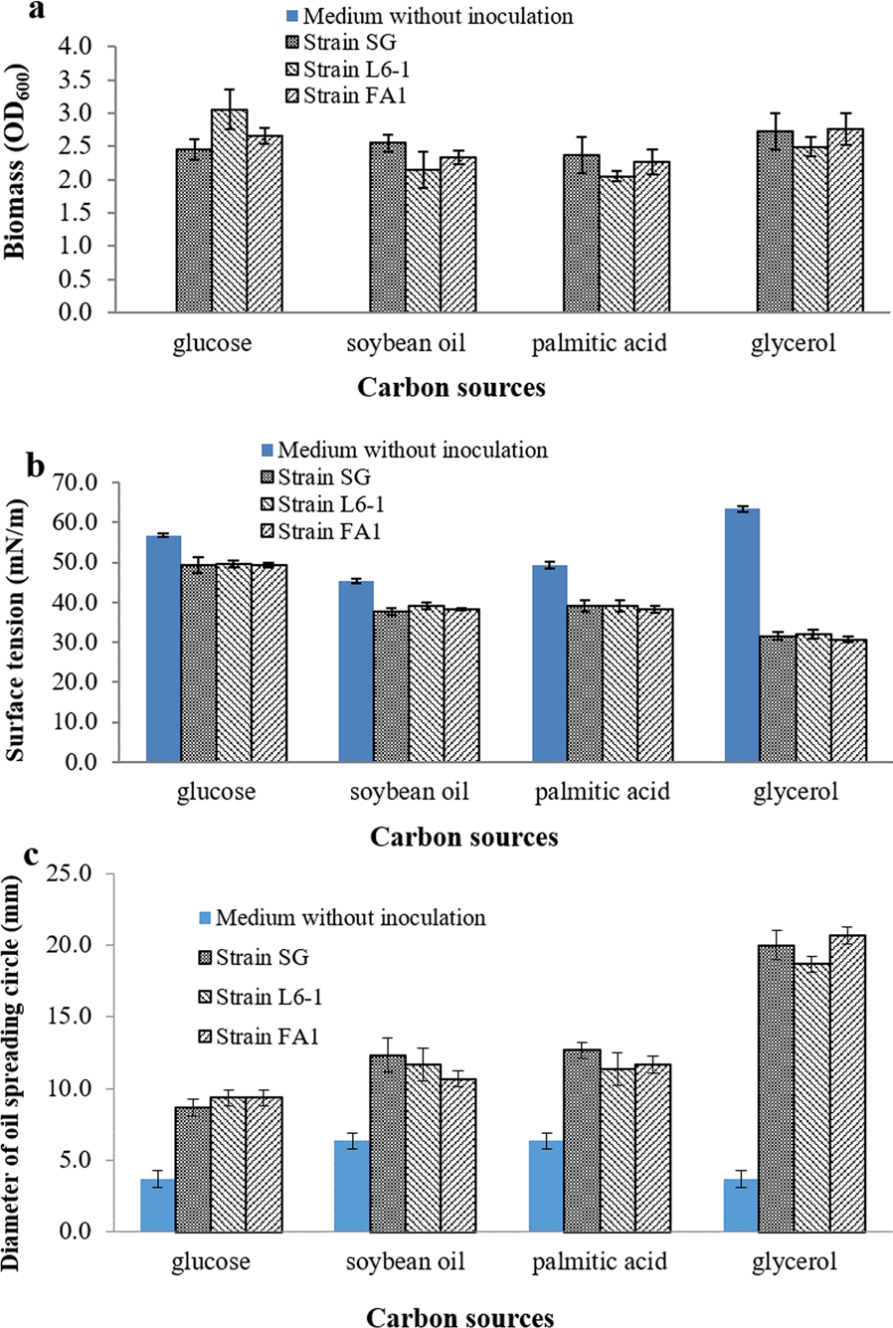
Anaerobic growth and production of rhamnolipids by *P. aeruginosa* strains using different carbon sources: **a** biomass (OD_600_), **b** surface activity and **c** oil spreading activity. Strain *P. aeruginosa* SG was used as the positive control

Using the tested carbon sources, the biomass (OD_600_), surface activity and oil spreading activity were shown in Fig. 2. Using different carbon sources, three *P. aeruginosa* strains obtained biomass with OD_600_ values between 1.80 and 4.00 under anaerobic conditions (Fig. 2a). Results indicated that three *P. aeruginosa* strains can anaerobically grow well using all the tested carbon sources. Results also confirmed that *P. aeruginosa* are facultative anaerobic bacteria [7, 16, 17]. Among the tested carbon sources, glucose and glycerol were more favorable to the anaerobic growth of *P. aeruginosa*. As water-soluble substrates, glucose and glycerol are more conducive to be rapid absorbed and metabolized. As shown in Fig. 2b, the surface tension of anaerobic culture was decreased to lower than 35 mN/m only using glycerol (from 63 mN/m to about 32 mN/m). Using glycerol, *P. aeruginosa* strains reduced the surface tension of anaerobic culture with the greatest decrease (49.5%). And the surface tension values were lowest. As shown in Fig. 2c, the diameters of oil spreading circles formed by anaerobic culture of three strains were greater than 15 mm when using glycerol as carbon source. Using other carbon sources, the forming oil spreading circles with diameters smaller than 10 mm. The formed oil spreading circles diameters can be used to indirectly determine biosurfactants concentration in bacterial culture [22]. Using glycerol, the oil spreading circles diameters formed by three *P. aeruginosa* strains, SG, L6-1 and FA1, were 20mm, 18mm and 21mm, respectively (Fig. 2c). Three *P. aeruginosa* strains, SG, L6-1 and FA1, produced 228 mg/l, 195 mg/l and 244 mg/l of rhamnolipids using glycerol under anaerobic conditions. Results were compared with single factor analysis of variance using software SPSS. Regarding to all three *P. aeruginosa* strains, the effect of carbon sources on anaerobic production of rhamnolipids was significant (*P* < 0.01). Using glucose, palmitic acid or soybean oil as carbon sources, the effect of strains on anaerobic production of rhamnolipids was not significant (*P* > 0.1). But the effect of strains on anaerobic production of rhamnolipids was significant (*P* < 0.05) when using glycerol as carbon sources. Results demonstrated that *P. aeruginosa* strains can produce rhamnolipids anaerobically when using glycerol as carbon source. Strain specificity resulted in the different anaerobic yield of rhamnolipids when using glycerol as carbon source.

Although all the tested substrates can be used for rhamnolipids production by *P. aeruginosa* under aerobic conditions [23–25], only glycerol can be used for anaerobic production of rhamnolipids by *P. aeruginosa*. Previous studies reported that strain *P. aeruginosa* SG can anaerobically produce rhamnolipids using glycerol [8, 21]. In this study, except for strain SG, other *P. aeruginosa* strains, L6-1 and FA1, can also anaerobically produce rhamnolipids using glycerol. Results confirmed that hypothesis 2 is credible. The specific substrate, glycerol, make *P. aeruginosa* strains achieve the anaerobic production of rhamnolipids.

### The effect of carbon sources concentrations on anaerobic production of rhamnolipids

**Fig. 3:**
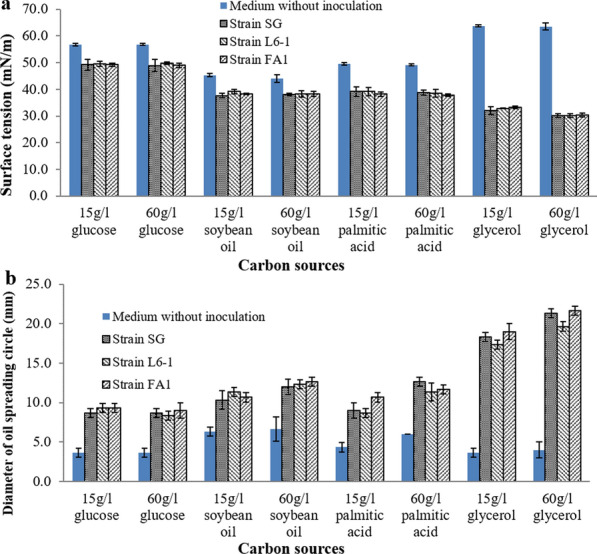
Effect of different carbon sources concentrations on anaerobic production of rhamnolipids by *P. aeruginosa* strains: **a** surface activity and **b** oil spreading activity

As shown in Fig. 3a, using glycerol with both concentrations of 15 g/l and 60 g/l, all three *P. aeruginosa* strains reduced the surface tension of anaerobic culture with the greatest decrease, from 63 mN/m to lower than 32 mN/m. However, using other carbon sources at both concentrations of 15 g/l and 60 g/l, the surface tension of anaerobic culture was decreased slightly. As shown in Fig. 3b, the diameters of oil spreading circles formed by three strains were greater than 15 mm when using 15 g/l and 60 g/l of glycerol. Using other carbon sources at both concentrations of 15 g/l and 60 g/l, the forming oil spreading circles with diameters smaller than 10 mm. Results once again confirmed that three *P. aeruginosa* strains can anaerobically produce rhamnolipids using glycerol as carbon source. Although other carbon sources with different concentrations were used, the tested carbon sources can not support anaerobic production of rhamnolipids by three *P. aeruginosa* strains yet.

Results of the present study demonstrated that glycerol was the only carbon source that supported anaerobic production of rhamnolipids by *P. aeruginosa*. The hypothesis 2 is correct. Glycerol substrate rather than the strain specificity contributed to the anaerobic production of rhamnolipids by *P. aeruginosa*.

### Anaerobic production of rhamnolipids using different nitrogen sources

**Fig. 4:**
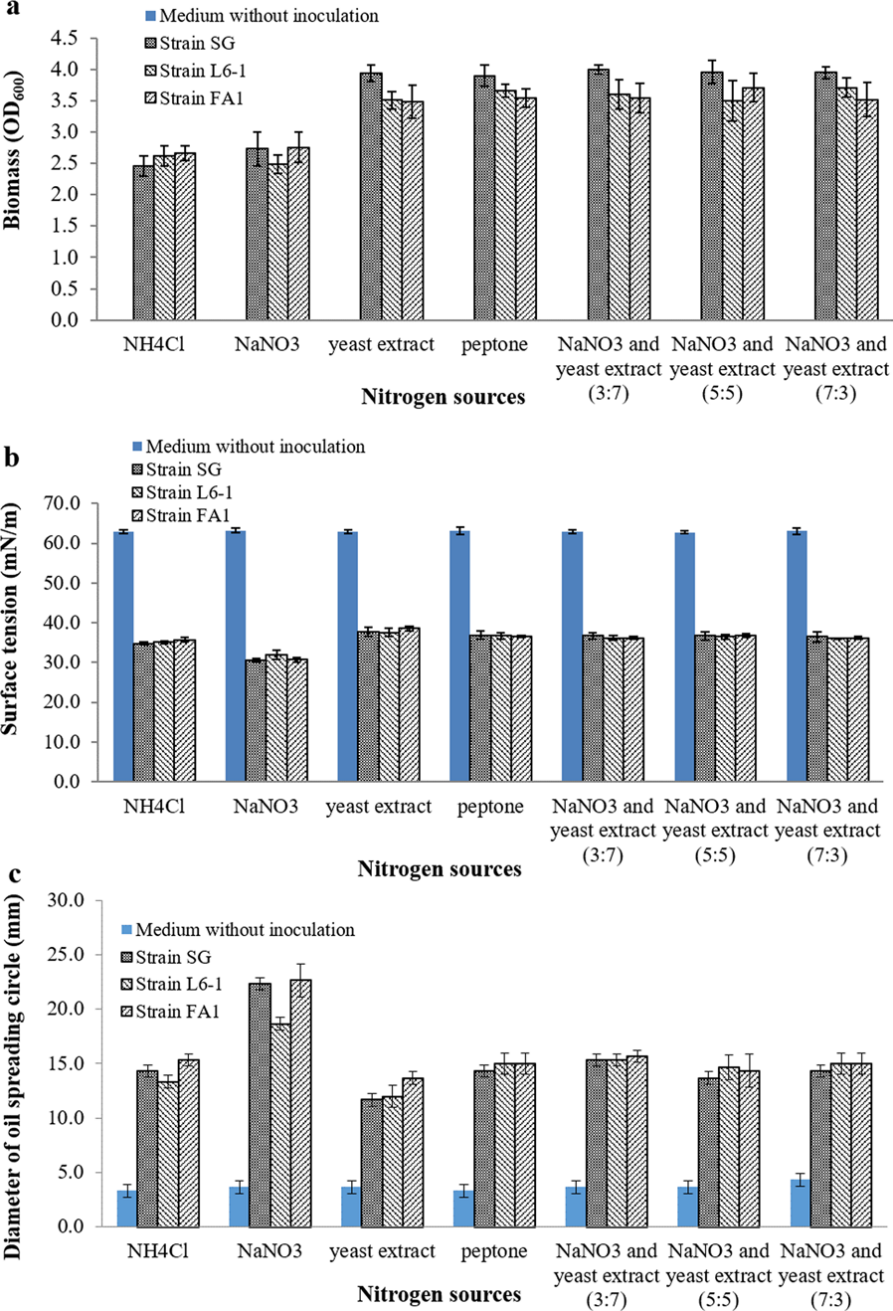
Anaerobic growth and production of rhamnolipids by *P. aeruginosa* strains using different nitrogen sources: **a** biomass (OD_600_), **b** surface activity and **c** oil spreading activity

Using the tested nitrogen sources, the biomass (OD_600_), surface activity and oil spreading activity were shown in Fig. 4. Under anaerobic conditions and using glycerol as carbon source, three *P. aeruginosa* strains obtained biomass with OD_600_ values between 2.50 and 4.50 using different nitrogen sources (Fig. 4a). Three *P. aeruginosa* strains anaerobically grew well using different nitrogen sources. As shown in Fig. 4a, the organic nitrogen sources were more favorable to the anaerobic growth of *P. aeruginosa* strains. The small molecular peptides contained in yeast extract and peptone may provide abundant and available nutrition for cell growth of *P. aeruginosa* [26]. As shown in Fig. 4b, the surface tension of anaerobic culture was reduced from 63 mN/m to lower than 40 mN/m using all tested nitrogen sources. Using glycerol as carbon source, *P. aeruginosa* strains anaerobically produced rhamnolipids using the tested nitrogen sources. Results also showed that glycerol is the key factor for anaerobic production of rhamnolipids by *P. aeruginosa*. Using NaNO_3_ as nitrogen source, *P. aeruginosa* strains reduced the surface tension of anaerobic culture to the lowest surface tension (31 mN/m), with the greatest decrease (51.3%). The oil spreading activity were shown in Fig. 4c. Using NaNO_3_ as nitrogen source, anaerobic culture of *P. aeruginosa* strains formed oil spreading circles with diameters about 20 mm. Results demonstrated that nitrate was a favorable nitrogen source for anaerobic production of rhamnolipids by *P. aeruginosa* when using glycerol as carbon source.

Although *P. aeruginosa* strains obtained higher biomass using the complex nitrogen sources of NaNO_3_ and yeast extract (Fig. 4a), the anaerobic production of rhamnolipids were relatively less (Fig. 4c). The organic nitrogen source may provide abundant and available nutrition for cell growth [26]. But studies also reported that organic nitrogen sources were not conducive to synthesize rhamnolipids [27]. Rhamnolipids is one secondary metabolite. Nitrate was reported to be the best nitrogen source for aerobic production of rhamnolipids [28, 29]. In this study, nitrate was also the excellent nitrogen source for anaerobic production of rhamnolipid. *P. aeruginosa* can grow and metabolize at both aerobic and anaerobic conditions [16, 17]. Because *P. aeruginosa* can use other electron acceptors except oxygen, such as nitrate [7, 18]. Nitrate was one good electron acceptors for anaerobic metabolism of *P. aeruginosa*.

### FTIR spectra analysis of anaerobically produced rhamnolipids

**Fig. 5:**
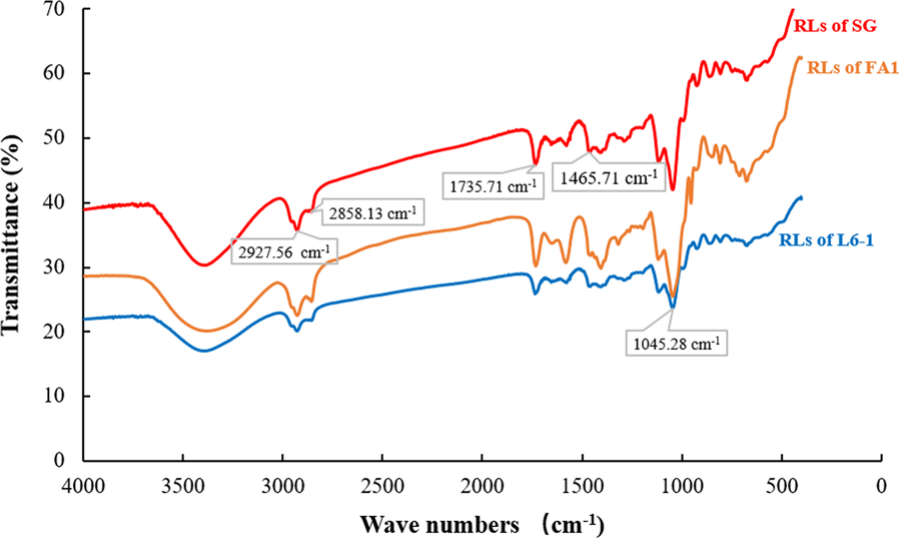
Fourier Transform infrared (FTIR) spectra analysis of anaerobically produced rhamnolipids from *P. aeruginosa* strains using glycerol and nitrate. Anaerobically produced rhamnolipids by strain SG was used as the positive control

The anaerobically produced rhamnolipids from *P. aeruginosa* strains using glycerol were confirmed by FT-IR spectra analysis, respectively. Rhamnolipids produced by *P. aeruginosa* SG was used as the positive control. As shown in Fig. 5, the FTIR spectra of the anaerobically produced rhamnolipids from *P. aeruginosa* L6-1 and FA1 were similar to that of rhamnolipids produced from strain SG. The FTIR spectra of the anaerobically produced rhamnolipids from *P. aeruginosa* strains were also similar to that of reported rhamnolipids [30, 31]. All the FTIR spectra had the characteristic absorption bands of rhamnolipids. Absorption bands around 2927 cm^− 1^, 2858 cm^− 1^ and 1465 cm^− 1^ were caused by the C-H stretching vibrations of aliphatic groups. The absorption bands around 1735 cm^− 1^ was caused by the ester groups. All the FTIR spectra also had the absorption area between 1452 cm^− 1^ and 1045 cm^− 1^ causing by the C–H and O–H vibrations. These are typical vibrations for carbohydrates. Results showed that *P. aeruginosa* strains did anaerobically produce rhamnolipids when using glycerol as carbon source.

### Activity of anaerobically produced rhamnolipids using glycerol

The rhamnolipids-water solutions (200 mg/l) were prepared using the extracted rhamnolipids products. All the anaerobically produced rhamnolipids using glycerol decreased the air-water surface tension from 72.6 mN/m to lower than 29 mN/m. Studies reported the aerobically produced rhamnolipids can decrease the air-water surface tension to lower than 27 mN/m [32]. The anaerobically produced rhamnolipids from *P. aeruginosa* exhibited excellent surface activity as well. Good surface activity helps to change contact angle, increase wetting activity and even facilitate wetting reversal. The excellent surface activity can also improve the capillary effect and facilitate the flow of groundwater or oil in porous media. These are of great significance for enhanced oil recovery and pollution remediation [33]. Anaerobically produced rhamnolipids using glycerol also showed better emulsifying activity to crude oil with EI_24_ values higher than 65%. Studies reported the aerobically produced rhamnolipids can emulsified crude oil with EI_24_ values ranging from 53 to 90% [32, 34]. The anaerobically produced rhamnolipids from *P. aeruginosa* also exhibited good emulsifying activity, which would be significant for enhanced oil recovery and pollution remediation [4, 35, 36]. The good emulsifying activity of biosurfactants can assist oil dispersion in oil reservoir and reduce oil viscosity [37]. Besides, the excellent emulsification effect can increase the solubility and the bioavailability of hydrophobic pollutants [36, 38].

### Glycerol intermediates and crude glycerol for anaerobic production of rhamnolipids

**Fig. 6:**
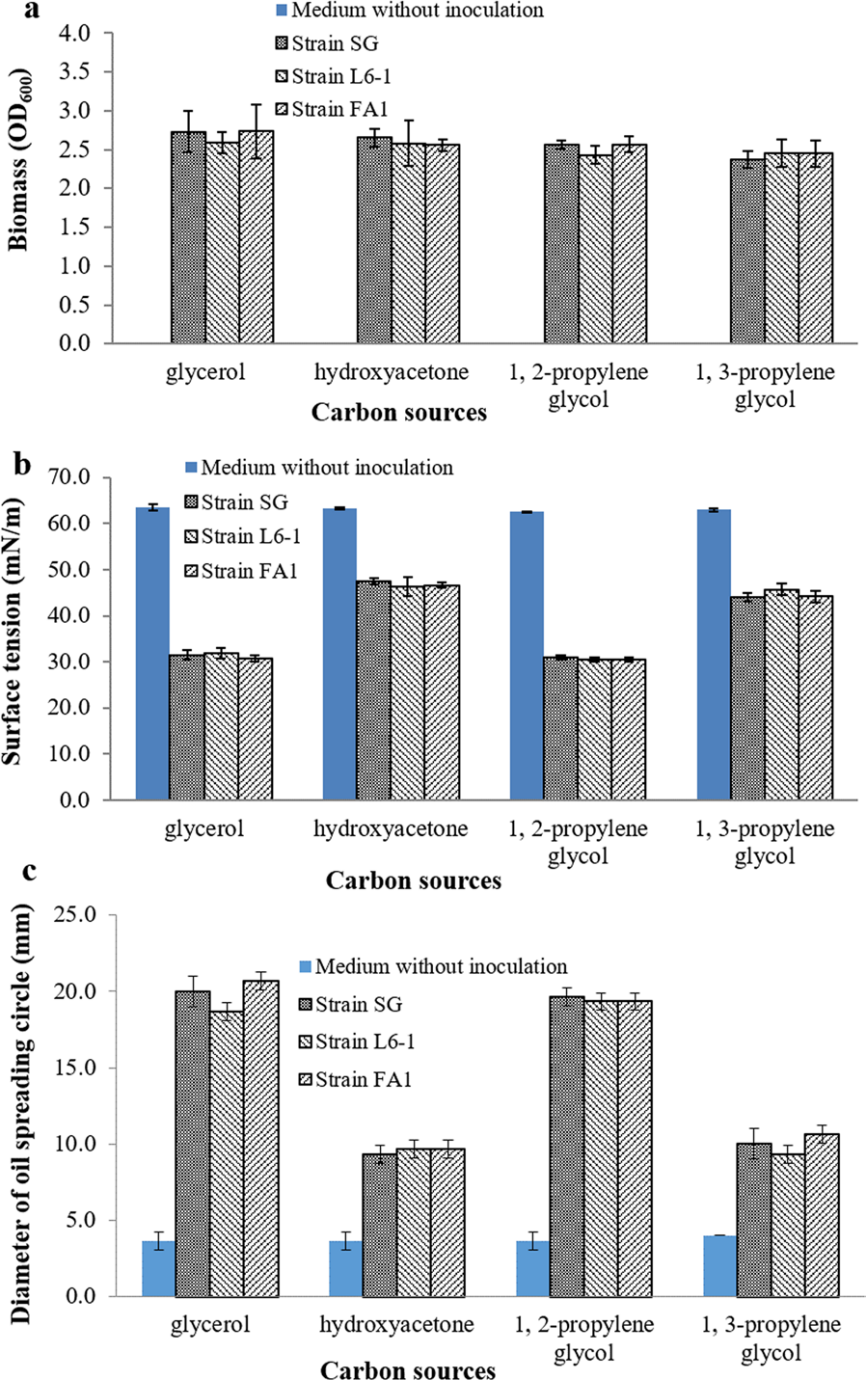
Anaerobic production of rhamnolipids by *P. aeruginosa* strains using crude glycerol

Glycerol is water soluble and easy to be absorbed by microorganisms. In the present study, only glycerol support *P. aeruginosa* to anaerobically produce rhamnolipids. Did the possible glycerol intermediates, hydroxyacetone, 1, 2-propylene glycol and 1, 3-propylene glycol, support for the anaerobic production of rhamnolipids by *P. aeruginosa*? As shown in Fig. 6, P. *aeruginosa* strains can anaerobically grow well using the possible glycerol intermediates. These substrates are involved in the glycolysis and gluconeogenesis pathways, which may be conducive to be rapidly absorbed and anaerobically metabolized by *P. aeruginosa* [39]. *P. aeruginosa* strains obtained the biomass with OD_600_ values between 2.00 and 3.00 (Fig. 6a). Merely 1, 2-propylene glycol supported *P. aeruginosa* strains to anaerobically produce rhamnolipids, reducing the surface tension of anaerobic culture to about 31 mN/m (Fig. 6b). Using 1, 2-propylene glycol, the oil spreading circles diameters formed by anaerobic culture were about 20 mm (Fig. 6c).

Results demonstrated that 1, 2-propylene glycol, similar to glycerol, can make *P. aeruginosa* to anaerobically biosynthesize rhamnolipids. Hauser and Karnovsky cultured *P. aeruginosa* strain using ^14^C-labeled glycerol-α-^14^C and glycerol-β-^14^C under aerobic conditions. And they found that the C_6_ unit of rhamnose group in rhamnolipids product was directly condensed from the two molecules glycerol (C_3_ unit) without carbon chain rearrangement [40]. The polyol can be oxidized into dihydroxyacetone (DHA). DHA can be converted into dihydroxyacetone phosphate (DHAP) which is involved in glycolysis and gluconeogenesis pathways [39, 41]. Then glycolysis and gluconeogenesis pathways are connected with the de novo synthesis of fatty acids. Therefore, *P. aeruginosa* can metabolize glycerol and 1, 2-propylene glycol to biosynthesize both the two precursors of rhamnolipids, TDP-L-rhamnose and β-hydroxy fatty acids. Glycerol metabolism facilitate the anaerobic biosynthesis of rhamnolipids in *P. aeruginosa*.

**Fig. 7:**
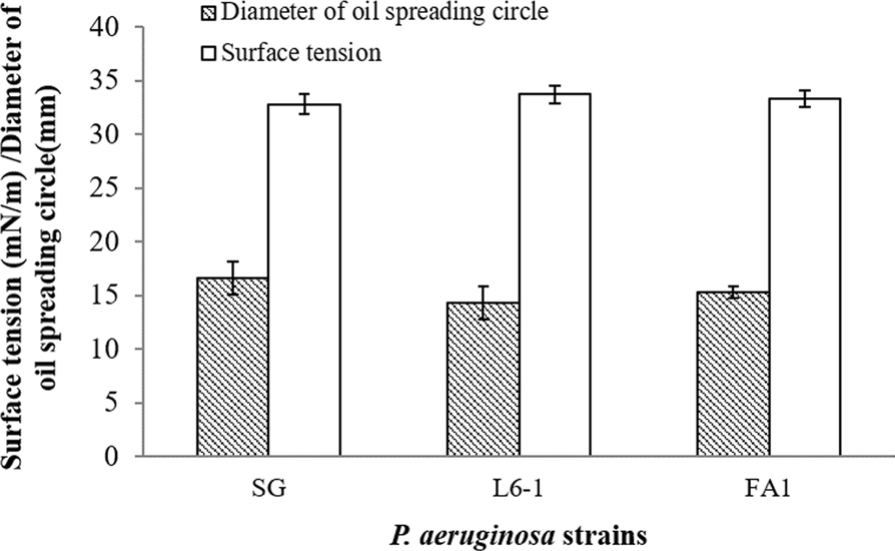
Anaerobic growth and production of rhamnolipids by *P. aeruginosa* strains using the possible metabolic intermediates and analogues of glycerol as carbon sources: **a** biomass (OD_600_), **b** surface activity and **c** oil spreading activity. Glycerol was used as the positive control

As shown in Fig. 7, three *P. aeruginosa* strains anaerobically produced rhamnolipids using crude glycerol. The surface tension of anaerobic culture was decreased to below 35 mN/m. The oil spreading circles diameters formed by anaerobic culture using crude glycerol were about 15 mm. Results showed that crude glycerol can be used for anaerobic production of rhamnolipids by *P. aeruginosa* strains as well. Previous studies reported aerobic production of rhamnolipids by *P. aeruginosa* using crude glycerol as low-cost substrate [42, 43]. In the present study, three *P. aeruginosa* strains achieved producing rhamnolipids anaerobically when using crude glycerol as substrates.

### Prospects and bottlenecks to anaerobic production of rhamnolipids

Although *P. aeruginosa* and its rhamnolipids have been extensively studied, studies on anaerobic production of rhamnolipids by *P. aeruginosa* are relatively less. Only a few studies reported that some rare *P. aeruginosa* strains can produce rhamnolipids anaerobically. The decisive factors for anaerobic production of rhamnolipids were unknown. Two possible hypotheses on the decisive factors for anaerobic production of rhamnolipids were proposed, the strains specificity of rare *P. aeruginosa* (hypothesis 1) and the effect of specific substrates (hypothesis 2). This study answered hypothesis 2 is credible. Glycerol substrate rather than the strain specificity contributed to the anaerobic production of rhamnolipids by *P. aeruginosa*. *P. aeruginosa* strains achieved producing rhamnolipids anaerobically when using glycerol or crude glycerol as substrates. Strain specificity resulted in the different anaerobic yield of rhamnolipids. Results are helpful to advance the research on anaerobic production of rhamnolipids, deepen the rhamnolipids biosynthesis theory and guide the optimization process for anaerobic production of rhamnolipids.

Anaerobic production of rhamnolipids can meet the in situ applications in anoxic environments, such as oil reservoirs, deep soil, sediments [7–9]. Based on anaerobic production of rhamnolipids, fermentation without aeration can avoid the foam problem in rhamnolipids production [14, 15]. The anaerobic production of rhamnolipids has important research and application significance.

But the anaerobic yield of rhamnolipids from *P. aeruginosa* using glycerol were lower than the aerobic yield of rhamnolipids [9]. The low anaerobic yield of rhamnolipids would limit its applications. Enhancing the anaerobic production of rhamnolipids is in demand. The anaerobic biosynthesis mechanism of rhamnolipids is significant for metabolic engineering of *P. aeruginosa* to enhance anaerobic yield. The biosynthesis pathways and key genes involved in anaerobic production of rhamnoipids have been revealed [44]. The anaerobic yield of rhamnolipids by *P. aeruginosa* can be enhanced by medium optimization, pathways regulation and genes modification.

Compared with anaerobic conditions, aerobic conditions are conducive to biosynthesize reducing power (NADPH or NADH_2_), which promotes fatty acid synthesis and ultimately enhances rhamnolipids synthesis. Increasing the reducing power (NADPH or NADH_2_) level will also probably enhance the anaerobic production of rhamnolipids by *P. aeruginosa.* β-hydroxy fatty acids are one of two key precursors in rhamnolipids biosynthesis. β-hydroxy fatty acids are derived from the de novo synthesis of fatty acids and β-oxidation of fatty acids [45]. Fatty acids metabolism requires a lot of reducing power. The reducing power maybe a limiting factor for anaerobic metabolism of fatty acids. In this study, glycerol is a more reductive carbon source than other tested glucose. Using glycerol as carbon source is conducive to reducing power synthesis for *P. aeruginosa*, which promotes fatty acids metabolism and rhamnolipids production under anaerobic conditions.

## Conclusions

In this study, two possible hypotheses on the decisive factors for anaerobic production of rhamnolipids were proposed, the strains specificity of rare *P. aeruginosa* (hypothesis 1) and the effect of specific substrates (hypothesis 2). Glycerol supported all tested *P. aeruginosa* strains to produce rhamnolipids anaerobically. Hypothesis 2 is credible. Glycerol substrate rather than the strain specificity contributed to the anaerobic production of rhamnolipids by *P. aeruginosa*. Strain specificity resulted in the different anaerobic yield of rhamnolipids. Nitrate was the excellent nitrogen source for anaerobic production of rhamnolipids. FTIR spectra analysis confirmed the anaerobically produced rhamnolipids by three *P. aeruginosa* strains using glycerol. Anaerobically produced rhamnolipids exhibited good surface activity and emulsifying activity. *P. aeruginosa* also produced rhamnolipids anaerobically using 1, 2-propylene glycol and crude glycerol. Prospects and bottlenecks to anaerobic production of rhamnolipids were also discussed. Results are helpful to advance the research on anaerobic production of rhamnolipids.

## Data Availability

The datasets supporting the conclusions of this article are included within the article.

## References

[1] Müller MM, Kügler JH, Henkel M, Gerlitzki M, Hörmann B, Pöhnlein M, Syldatk C, Hausmann R (2012). Rhamnolipids-next generation surfactants?. J Biotechnol.

[2] Varjani S, Upasani VN (2017). Critical review on biosurfactant analysis, purification and characterization using rhamnolipid as a model biosurfactant. Bioresour Technol.

[3] Chong H, Li Q (2017). Microbial production of rhamnolipids: opportunities, challenges and strategies. Microb Cell Fact.

[4] Gudiña EJ, Rodrigues AI, Alves E, Domingues MR, Teixeira JA, Rodrigues LR (2015). Bioconversion of agro-industrial by-products in rhamnolipids toward applications in enhanced oil recovery and bioremediation. Bioresour Technol.

[5] Cameotra SS, Singh P (2009). Synthesis of rhamnolipid biosurfactant and mode of hexadecane uptake by *Pseudomonas* species. Microb Cell Fact.

[6] Zou H, Du W, Ji M, Zhu R (2016). Enhanced electrokinetic remediation of pyrene-contaminated soil through pH control and rhamnolipids addition. Environ Eng Sci.

[7] Domingues PM, Almeida A, Leal LS (2017). Bacterial production of biosurfactants under microaerobic and anaerobic conditions. Rev Environ Sci Biotechnol.

[8] Zhao F, Zhang J, Shi R, Han S, Ma F, Zhang Y (2015). Production of biosurfactant by a *Pseudomonas aeruginosa* isolate and its applicability to *in situ* microbial enhanced oil recovery under anoxic conditions. RSC Adv.

[9] Zhao F, Shi R, Ma F (2018). Oxygen effects on rhamnolipids production by *Pseudomonas aeruginosa*. Microb Cell Fact.

[10] Câmara J, Sousa M, Neto ELB (2019). Application of rhamnolipid biosurfactant produced by *Pseudomonas aeruginosa *in microbial-enhanced oil recovery (MEOR). J Pet Explor Prod Technol.

[11] Youssef NH, Simpson DR, McInerney MJ (2013). In-situ lipopeptide biosurfactant production by *Bacillus* strains correlates with improved oil recovery in two oil wells approaching their economic limit of production. Int Biodeterior Biodegrad.

[12] Zhao F, Li P, Guo C, Shi R, Zhang Y (2018). Bioaugmentation of oil reservoir indigenous *Pseudomonas aeruginosa* to enhance oil recovery through in-situ biosurfactant production without air injection. Bioresour Technol.

[13] Albino JD, Namb IM (2010). Partial characterization of biosurfactant produced under anaerobic conditions by *Pseudomonas* sp ANBIOSURF-1. Adv Mater Res.

[14] Anic I, Apolonia I, Franco P (2018). Production of rhamnolipids by integrated foam adsorption in a bioreactor system. AMB Expr.

[15] Long X, Shen C, He N (2017). Enhanced rhamnolipids production via efficient foam-control using stop valve as a foam breaker. Bioresour Technol.

[16] Arai H (2011). Regulation and function of versatile aerobic and anoxic respiratory metabolism in *Pseudomonas aeruginosa*. Front Microbiol.

[17] Schobert M, Jahn D (2010). Anaerobic physiology of *Pseudomonas aeruginosa* in the cystic fibrosis lung. Int J Med Microbiol.

[18] Chayabutra C, Wu J, Ju LK (2001). Rhamnolipids production by *Pseudomonas aeruginosa* under denitrification: effects of limiting nutrients and carbon substrates. Biotechnol Bioeng.

[19] Shekhar S, Sundaramanickam A, Balasubramanian T (2015). Biosurfactant producing microbes and their potential applications: a review. Crit Rev Environ Sci Technol.

[20] Zhao F, Shi R, Zhao J (2015). Heterologous production of *Pseudomonas aeruginosa* rhamnolipid under anaerobic conditions for microbial enhanced oil recovery. J Appl Microbiol.

[21] Zhao F, Zhou J, Han S, Ma F, Zhang Y, Zhang J (2016). Medium factors on anaerobic production of rhamnolipids by *Pseudomonas aeruginosa* SG and a simplifying medium for in situ microbial enhanced oil recovery applications. World J Microbiol Biotechnol.

[22] Zhao F, Liang X, Ban Y, Han S, Zhang J, Zhang Y, Ma F (2016). Comparison of methods to quantify rhamnolipids and optimization of oil spreading method. Tenside Surfact Det.

[23] Nicolò MS, Cambria MG, Impallomeni G, Rizzo MG, Pellicorio C, Ballistreri A, Guglielmino SP (2017). Carbon source effects on the mono/dirhamnolipid ratio produced by *Pseudomonas aeruginosa* L05, a new human respiratory isolate. New Biotechnol.

[24] Ehinmitola EO, Aransiola EF, Adeagbo OP (2018). Comparative study of various carbon sources on rhamnolipid production. S Afr J Chem Eng.

[25] Tan YN, Li Q (2018). Microbial production of rhamnolipids using sugars as carbon sources. Microb Cell Fact.

[26] Kerr ED, Schulz BL (2016). Vegemite Beer: yeast extract spreads as nutrient supplements to promote fermentation. PeerJ.

[27] Reis RS, Pereira AG, Neves BC, Freire DMG (2011). Gene regulation of rhamnolipid production in *Pseudomonas aeruginosa*—a review. Bioresour Technol.

[28] Soberón-Chávez G, Lepine F, Déziel E (2005). Production of rhamnolipids by *Pseudomonas aeruginosa*. Appl Microbiol Biotechnol.

[29] Reis RS, Rocha SLG, Chapeaurouge DA, Domont GB, Santa Anna LMM, Freire DMG, Perales J (2010). Effects of carbon and nitrogen sources on the proteome of *Pseudomonas aeruginosa* PA1 during rhamnolipid production. Process Biochem.

[30] Leitermann F, Syldatk C, Hausmann R (2008). Fast quantitative determination of microbial rhamnolipids from cultivation broths by ATR-FTIR Spectroscopy. J Biol Eng.

[31] Kiefer J, Radzuan MN, Winterburn J (2017). Infrared spectroscopy for studying structure and aging effects in rhamnolipid biosurfactants. Appl Sci.

[32] Nitschke M, Cost SGVAO, Contiero J (2005). Rhamnolipid surfactants: an update on the general aspects of these remarkable biomolecules. Biotechnol Prog.

[33] Park T, Jeon M, Yoon S (2019). Modification of interfacial tension and wettability in Oil–Brine–Quartz system by in situ bacterial biosurfactant production at reservoir conditions: implications for microbial enhanced oil recovery. Energy Fuels.

[34] Pekdemir T, Copur M, Urum K (2005). Emulsification of crude oil–water systems using biosurfactants. Process Saf Environ.

[35] Rahman KSM, Rahman TJ, Kourkoutas Y, Petsas I, Marchant R, Banat IM (2003). Enhanced bioremediation of n-alkane in petroleum sludge using bacterial consortium amended with rhamnolipids and micronutrients. Bioresour Technol.

[36] Varjani SJ, Upasani VN (2016). Core Flood study for enhanced oil recovery through ex-situ bioaugmentation with thermo-and halo-tolerant rhamnolipids produced by *Pseudomonas aeruginosa* NCIM 5514. Bioresour Technol.

[37] Hosseininoosheri P, Lashgari HR, Sepehrnoori K (2016). A novel method to model and characterize in-situ bio-surfactant production in microbial enhanced oil recovery. Fuel.

[38] Sponza DT, Gök O (2010). Effect of rhamnolipids on the aerobic removal of polyaromatic hydrocarbons (PAHs) and COD components from petrochemical wastewater. Bioresour Technol.

[39] Pobletecastro I, Wittmann C, Nikel PI (2020). Biochemistry, genetics and biotechnology of glycerol utilization in *Pseudomonas* species. Microb Biotechnol.

[40] Hauser G, Karnovsky ML (1957). Rhamnose and rhamnolipide biosynthesis by *Pseudomonas aeruginosa*. J Biol Chem.

[41] Biebl H, Menzel K, Zeng AP, Deckwer WD (1999). Microbial production of 1,3-propanediol. Appl Microbiol Biotechnol.

[42] Eraqi WA, Yassin AS, Ali AE (2016). Utilization of crude glycerol as a substrate for the production of rhamnolipid by *Pseudomonas aeruginosa*. Biotechnol Res Int.

[43] Zhao F, Jiang H, Sun H (2019). Production of rhamnolipids with different proportions of mono-rhamnolipids using crude glycerol and a comparison of their application potential for oil recovery from oily sludge. RSC Adv.

[44] Zhao F, Wang Q, Zhang Y (2021). Anaerobic biosynthesis of rhamnolipids by *Pseudomonas aeruginosa*: performance, mechanism and its application potential for enhanced oil recovery. Microb Cell Fact.

[45] Abdel-Mawgoud AM, Lépine F, Déziel E (2014). A stereospecific pathway diverts β-oxidation intermediates to the biosynthesis of rhamnolipid biosurfactants. Chem Biol.

